# Reversed gender ratio of autism spectrum disorder in Smith-Magenis syndrome

**DOI:** 10.1186/s13229-017-0184-2

**Published:** 2018-01-08

**Authors:** Heidi Elisabeth Nag, Ann Nordgren, Britt-Marie Anderlid, Terje Nærland

**Affiliations:** 1 Frambu Resource Centre for Rare Disorders, Siggerud, Norway; 2 University of Stavanger, Stavanger, Norway; 3 Karolinska Centre for Rare Diseases, Karolinska University Hospital, Solna, Sweden; 4 NevSom, Department of Rare Disorders and Disabilities, Oslo University Hospital, Oslo, Norway; 5 KG Jebsen Centre for Psychosis Research, NORMENT, University of Oslo, Oslo, Norway

**Keywords:** Gender, Autism symptomatology, Smith-Magenis syndrome

## Abstract

**Background:**

A substantial amount of research shows a higher rate of autistic type of problems in males compared to females. The 4:1 male to female ratio is one of the most consistent findings in autism spectrum disorder (ASD).

Lately, the interest in studying ASD in genetic disorders has increased, and research has shown a higher prevalence of ASD in some genetic disorders than in the general population.

Smith-Magenis syndrome (SMS) is a rare and complex genetic syndrome caused by an interstitial deletion of chromosome 17p11.2 or a mutation on the retinoic acid induced 1 gene. The disorder is characterised by intellectual disability, multiple congenital anomalies, obesity, neurobehavioural abnormalities and a disrupted circadian sleep-wake pattern.

**Methods:**

Parents of 28 persons with SMS between 5 and 50 years old participated in this study. A total of 12 of the persons with SMS were above the age of 18 at the time of the study. A total of 11 came from Sweden and 17 were from Norway.

We collected information regarding the number of autism spectrum symptoms using the Social Communication Questionnaire (SCQ) and the Social Responsiveness Scale (SRS). Adaptive behaviour was also measured using the Vineland Adaptive Behavior Scale II. The level of intellectual disability was derived from a review of the medical chart.

**Results:**

We found significant gender differences in ASD symptomatology using the SCQ and SRS questionnaires. We found approximately three females per male above the SCQ cutoff. The same differences were not found in the intellectual level and adaptive behaviour or for behavioural and emotional problems.

Gender had an independent contribution in a regression model predicting the total SCQ score, and neither the Vineland Adaptive Behavior Scale II nor the Developmental Behaviour Checklist had an independent contribution to the SCQ scores.

**Conclusion:**

We found a clear reversed gender difference in ASD symptomatology in persons with SMS. This may be relevant in the search for female protective factors assumed to explain the male bias in ASD.

## Background

A substantial amount of research shows a higher rate of autistic type problems in males compared to females. The 4:1 male to female ratio is one of the most consistent findings in autism spectrum disorder (ASD) research [1–3], and a gender difference has been a part of the description of ASD since the first characterisation of the disorders.

ASD occurs in conditions with X-linked recessive inheritance, but because of the rarity of these disorders, this inheritance cannot explain the male bias in prevalence of ASD [4]. The fact that most ASD risk loci are found in autosomal regions makes the male bias in ASD largely unexplained [5]. Most current data suggest that the male bias is more likely to be due to female protective factors rather than male-specific risk factors, but comprehensive molecular explanations are lacking for both [6].

Gender ratios in ASD differ substantially from study to study. Among individuals with ASD and normal cognitive functioning, gender differences as high as 9:1 have been reported [7]. A newer systematic review and meta-analysis from Loomes et al. [8] found a male to female ratio closer to 3:1 than 4:1. According to Loomes et al. [8], the main reasons for this change were both how ASD was diagnosed and what population were used to investigate the male to female ratio in ASD in different studies. Loomes [8] found that studies screening the general populations for ASD had a lower male to female ratio than studies investigating population with pre-existing diagnosis. In cohorts with ASD in combination with intellectual disability, the ratio varies between 2:1–7:1 [2, 4]. Loomes et al. [8] also found a lower male to female ratio in their meta-analysis in the subgroup of the studies including participants with lower IQ. Epidemiological studies describe the degree of intellectual disability and the ascertainment approach as major explanations behind the varying ratios that were reported [9].

The particular biological aetiologies of autistic problems are probably also relevant, even when the degree of intellectual disability (ID) is controlled for, but such a line of investigation has not yet been explored. How different biological pathways to ASD differ in the ASD-gender ratio may shed light on basic ASD biology.

ASD is in the Diagnostic and Statistical Manual V (DSM V) referred to as a dyad of impairments; difficulties in social interactions and social communications; and restricted and repetitive behaviour, interests, and activities [10]. Gender differences in profiles of autistic symptoms have a limited research base [11]. Several studies [11–13] have found that males have more restricted and repetitive behaviours than females. Some studies have found that females have more impairment in social reciprocity and communication than males, but these findings are not consistent [13]; others have found that females with ASD have better sociability skills than males with ASD [14].

Lately, the interest in studying ASD in genetic disorders has increased, and research shows a higher prevalence of ASD in some genetic disorders than in the general population [15]. The focus so far has been on the prevalence and phenomenology in different syndromes, and further studies are required to tell us more about the differences in ASD phenomenology between ASD in genetic syndromes and idiopathic autism. Using the Autism Screening Questionnaire (ASQ), Oliver et al. [16] found a high level of autism (> 45%) in individuals with Cornelia de Lange syndrome (CdLS) and fragile X syndrome (FXS) (only males with FXS participated in the study) but lower levels in individuals with cri du chat syndrome (CDCS), Angelman syndrome and Prader Willis syndrome (PWS) (< 20%). Individuals with Lowe syndrome and Smith-Magenis syndrome (SMS) were more in the middle with approximately 35% scoring above the cutoff for autism. No significant gender differences in any of the syndromes were found. Another study concerning tuberous sclerosis (TSC) found no significant differences between females and males regarding ASD [17]. Recently, Nærland et al. [18] published an article regarding gender differences in Down syndrome. The gender ratios in their sample were approximately 2M:1F, which is slightly less than expected in idiopathic ASD with the same degree of ID.

SMS is one of the rare disorders where ASD has been described as a prominent part of the disorder [19] but also a disorder where gender differences in ASD symptoms, favouring females, have been found. Laje et al. [19] found that females had higher *T* scores on the Social Responsiveness Scale (SRS) total and on the ‘Social cognition’ and ‘Autistic mannerisms’ subscales. In this study, they did not control for the gender differences already accounted for in the gender-specific norms. They did not find any significant gender differences in Social Communication Questionnaire (SCQ) scores [20].

SMS is a rare and complex genetic syndrome caused by an interstitial deletion of chromosome 17p11.2 [21] or a mutation on the retinoic acid induced 1 (RAI1) gene [22]. Most SMS patients have a deletion containing 76 genes [23], but the patients with mutations in the RAI1 gene also display most of the core features of SMS, which indicates that the RAI1 gene is a dosage-sensitive gene responsible for most of the symptoms in SMS patients [24]. The disorder is characterised by intellectual disability, multiple congenital anomalies, obesity, neurobehavioural abnormalities and a disrupted circadian sleep-wake pattern [25]. The incidence of SMS is estimated to range from 1:15,000–1:25,000 births [26]. Delayed diagnosis is common, although the use of array-CGH and SNP-array analyses in routine clinical practice, together with greater recognition of the syndrome in the last decade, has led to earlier diagnosis [27].

Children and adults with SMS appear to have unique neurobehavioural problems that are challenging for both parents and professionals. These problems include sleep disturbances, self-injurious and maladaptive behaviours, stereotypies, and sensory integration disorders [28]. A thorough investigation of aggressive behaviours of a cohort with SMS showed that self-injurious behaviour, physical aggression and destructive behaviour were all significantly more prevalent in persons with SMS compared with a cohort of persons with IDs of mixed aetiologies [29]. In this study, 96.9% of participants displayed self-injurious behaviour, 87.5% exhibited physical aggression, 81.3% showed destructive behaviour and 43.8% were verbally aggressive [29]. SRS scores consistent with ASD have also been identified in almost 90% of the investigated populations with SMS [19]. A progression of autistic-like behaviour has also been described in young children with SMS [20]. A study comparing several genetic disorders (PWS, FXS, CdLS, CDCS, etc.) found that persons with SMS scored higher (were more impaired) than PWS and CDCS in the social domain, but in the two other domains (communication and repetitive behaviour), they did not differ from the other groups [16].

In addition to the study by Laje [19] mentioned earlier, two other studies have looked at gender differences in SMS [19, 30]. In an animal model study, Huang et al. [24] found a sexually dimorphic phenotype regarding obesity in mice (females were significantly more obese than males) with loss of RAI1 functions, but due to high mortality, they did not investigate this any further and the cause of the sexually dimorphic phenotype is not clear. Edelman et al. [30] found some somatic differences between males and females such as myopia, cold hands and feet, eating/appetite problems and possible hypersensitivity (problems finding shoes to fit) in females; Edelman et al. also found that females had more frustration with communication than males.

Measuring ASD in genetic syndromes is fraught with some difficulties. Individuals with known genetic syndromes are usually excluded from the standardisation of ASD assessment tools, and it is known that degree of intellectual disability influences these tools [31]. Additionally, it is recently documented that the commonly used ASD assessment tools are highly influenced by parent-reported behavioural and emotional problems [32]. In the SMS population with its varying cognitive abilities and high rates of behavioural problems, it is therefore important to control for these factors when making claims about ASD symptomatology.

The main aim of this study was to investigate gender differences in rates and profile of ASD symptoms in SMS when controlling for rates of emotional and behaviour problems and adaptive behaviour as a proxy for developmental level. Based on previous research and our own clinical experiences, we hypothesised that the usual increased rate of ASD symptoms in males (the male bias) would be absent in a sample of individuals with SMS.

## Methods

### Recruitment and participants

This study was part of a larger assessment study of SMS in Norway and Sweden. The participants were recruited through Frambu Resource Centre for Rare Disorders (Frambu) and the Smith-Magenis Foundations in Norway and Sweden (both family support groups). Both organisations spread information regarding the study via their Facebook sites and email lists. Frambu, which is one of nine publicly funded centres of expertise administered by the Norwegian National Advisory Unit on Rare Disorders, has its own register, which is based on informed consent. Frambu could therefore send invitations to registered families with a child or an adult with a diagnosis of SMS. The Swedish families were recruited through the Swedish Smith-Magenis Foundation both through information via their Facebook site and through information at their annual gathering. The only inclusion criterion was a genetically confirmed diagnosis of SMS. The diagnosis was confirmed by review of the genetic testing reports. The parents and the patients above the age of 16 provided written consent to participate in the study.

Parents of 28 persons with SMS aged between 5 and 50 years participated in this study; 12 of the persons with SMS were above the age of 18 at the time of the study. A total of 11 came from Sweden and 17 were from Norway (all the Norwegian patients were recruited through Frambu). In Norway, we know of 36 patients diagnosed with SMS and in Sweden 20; we have thus included approximately 47% of the Norwegian and approximately 55% of the Swedish SMS population. In Norway, 58% (*n* = 21) are females and in Sweden, 50% (*n* = 10) are females.

The level of ID was derived from a review of the medical charts. Consents were given to collect medical charts from the paediatric/habilitation and pedagogical centres. The levels of ID were collected from these charts. There was a wide variety as to who administered the test, with what instrument and at what age the level of ID was established.

### Demographics

The demographics are displayed in Table 1. The mean age was 18.5 with a range from 5.1–50.5. The intellectual disability (ID) level was available from medical charts; seven of the patients did not have ID. It seems that more females had lower levels of ID, but this gender difference was not significant (asympt. *p* = 0.07).

**Table 1:** Demographics

	Total	Females	Males
*N*	28	15	13
Mean age	18.5	16.2	22.2
Range	5.1–50.5	5.1–33.9	5.1–50.5
Genetics			
Deletion	25	12	13
Mutation	3	3	0
ID grade			
No ID	7	3	4
Mild	5	1	4
Moderate	15	10	5
Severe/profound	1	1	0

### Instruments

The Social Communication Questionnaire (SCQ) is a standardised screening tool for ASD [33]. The SCQ was used to assess the number of autism symptoms [33]. The questionnaire is used from the age of four. It contains 40 items, which are answered with ‘Yes’ (= 1) or ‘No’ (= 0) and comes in two versions. SCQ-Current covers the individual’s behaviour during the most recent 3 months, whereas SCQ-Lifetime is based on the individual’s entire developmental history. Both versions give a single total score, where a score of 15 or above is regarded as an indicator of possible ASD. The SCQ are also scored in three different domains: the reciprocal social interaction domain, communication domain and repetitive domain. In this study, the SCQ-Lifetime questionnaire was used [34]. In the initial standardisation of the assessment tool, a good reliability was reported with a Cronbach’s alpha of 0.84–0.93 across the age groups and a Cronbach’s alpha of 0.81–0.92 across the diagnostic groups [33]. Rutter et al. [33] also measured the validity and found a correlation of 0.71 between SCQ and the Autism Diagnostic Interview-Revised (ADI-R). In two groups of children with Down syndrome (DS) with ASD and DS without ASD, Magyar et al. [35] investigated the validity of SCQ and found that it did discriminate between the two groups. Children with DS and ASD obtained a significantly higher total score on the SCQ than children with DS only. SCQ is used in research on different genetic disorders [35, 36] including SMS [19].

The Social Responsiveness Scale (SRS) is a 65-item, quantitative parent-reported or adult self-reported measure that assesses social impairment associated with ASD [37]. The SRS enquires about specific and observable elements of reciprocal social behaviour (39 items), social use of language (6 items) and behaviour characteristics of children with autism and other PDDs (20 items), and it generates a standardised score. In addition to a total score, SRS consists of five subscores: Social Awareness, Social Cognition, Social Communication, Social Motivation and Autistic Mannerisms. In the initial standardisation of the questionnaire, the reliability was tested across gender and parents’ and teachers’ reports and in clinical settings. A good reliability was reported across these groups with a Cronbach’s alpha of 0.93–0.97 [37]. The validity of the SRS has also been evaluated and a strong association between the SRS and the ADI-R was found [37]. Recently, in a large sample of idiopathic ASD, the SRS scores were shown to be influenced by rates of behavioural problems [32]. We therefore use both the SCQ and the SRS in this study and we assess the effect of behavioural problems. Since the SRS *T* score norms are different for males and females, we chose to use raw scores in addition to *T* scores when comparing the genders.

The Developmental Behaviour Checklist (DBC) [38, 39] is a questionnaire completed by parents or other primary caretakers or teachers that report problems over a 6-month period. Each behavioural description is scored on a 0, 1 and 2 rating where 0 = ‘not true as far as you know’, 1 = ‘somewhat or sometimes true’, and 2 = ‘very true or often true’. Five versions of the Checklist are available: the Parent/Carer version (DBC-P), the Teacher version (DBC-T), the Adult version (DBC-A), the Short-form (DBC-P24) and the Monitoring chart (DBC-M). In this study, the DBC-P was used.

The Vineland Adaptive Behavior Scale II (VABS II) [40, 41] is a semi-structured interview or rating form of the parents or caregivers that assesses the everyday behavioural functioning of children and adults from birth throughout life. In this study, both the interview form (Norwegian cohort) and the parent/caregiver rating form (Swedish cohort) were used. The scales yield standard scores (mean = 100: one standard deviation (SD) = 15) in the domains of communication, daily living skills, socialisation and motor function, as well as a total sum score on adaptive behaviour composite. Each domain contains several subdomains. Motor function can only be assessed in children less than 6 years of age. In this study, the Norwegian and Swedish versions of the scales based on Scandinavian normative data were used. VABS II is a standardised and validated tool. Many studies have confirmed its reliability and validity making this measure one of the most widely used assessment tools of adaptive behaviour [42]. This tool has also been used with SMS several times [43, 44].

The SRS, SCQ and DBC were all mailed to the parents after they consented to participate in the study. The parents filled in the information at home and mailed the questionnaire back to the researchers in a prepaid envelope. The VABS II were conducted in two different ways; the Norwegian cohort was interviewed on the telephone, and the Swedish cohort was mailed the parent/caregiver rating form together with the other questionnaires. The difference in procedure was due to language issues of performing the telephone interview with the Swedish cohort.

These instruments were chosen, instead of the gold-standard instruments ADI-R and Autism Diagnostic Observation Schedule (ADOS), because of their ease of use, because they have been used earlier with SMS, and to assess persons scattered around Norway and Sweden with the least possible burden for the patients.

### Statistical analysis

Data were compiled for statistical analysis using the Statistical Package for the Social Sciences (SPSS) version 23 (IBM). Analysis of group differences in the degree of ID was conducted with the Mann-Whitney independent sample test. Descriptive statistics were derived, and the total scores and subscores obtained from the SRS and the SCQ were analysed as continuous dependent variables using *t* tests. The ratio was calculated as number of females above the cutoff on the SCQ total score divided by the number of males above the cutoff. Effect sizes (Cohen’s *d*) were calculated using Social Science Statistics’ online resources. The two-sided Fisher’s exact test was used to test the proportion of males and females above the SCQ cutoff and in the different SRS classifications. Multiple regression analyses were conducted to assess the impact of ‘gender’, ‘DBC’ and ‘VABS II standard scores’ on the ‘total SCQ score’. The normality of the residuals was checked using the visual inspection of P-P plots. Due to the combination of dichotomous and continuous predictor variables, we report the standardised coefficients (*β*), in addition to unstandardized *B*.

## Results

### Social Communication Questionnaire

**Fig. 1:**
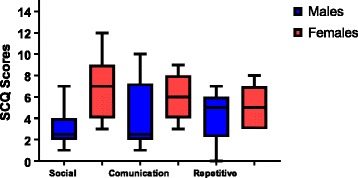
SCQ subdomain scores divided by males and females

The SCQ scores from 27 patients were analysed. A total of 52% scored above the cutoff (≥ 15). The females had higher scores on both the SCQ total score and all domains, but only the total SCQ score and the reciprocal social interaction domain showed a significant gender difference. A total of 25% of the males and 73% of the females scored above the ≥ 15 cutoff (*p* = 0.021). This provides a gender ratio of 3:1 and favours the females. All the SCQ scores are summarised in Table 2. The means for the males and females on each SCQ subdomain score are plotted in Fig. 1. How the ID grades are distributed between the males and females with SCQ scores above versus below the ASD cutoff are displayed in Table 3 (females) and Table 4 (males).

**Table 2:** Social Communication Questionnaire scores

	Total (*N* = 27^a^)	Females (*N* = 15)	Males (*N* = 12)	*p* value (Cohen’s *d*)
SCQ total (SD)	16.04 (6.10)	19.07 (4.77)	12.25 (5.55)	0.003 (1.32)
Reciprocal Social Interaction (SD)	5.19 (3.05)	6.87 (2.83)	3.08 (1.78)	0.000290 (1.60)
Communication (SD)	5.07 (2.73)	5.93 (1.98)	4.00 (3.22)	0.086 (0.72)
Repetitive behaviour (SD)	4.81 (2.19)	5.27 (1.91)	4.25 (2.45)	0.252 (0.46)
	*N*(%)[ratio]	*N*(%)	*N*(%)	
Number above cutoff (≥ 15)	14(52)[2.93]	11(73)	3(25)	0.021

^a^One parent did not return the SCQ questionnaire

**Table 3:** ID grade and SCQ-cutoff crosstabulation males

			SCQ-cutoff		Total
			≤ 15	≥ 15	
ID grade	No ID	Count	3	0	3
		% within ID grade	100.0	0.0	100.0
	Mild	Count	2	2	4
		% within ID grade	50.0	50.0	100.0
	Moderate	Count	4	1	5
		% within ID grade	80.0	20.0	100.0
Total		Count	9	3	12
		% within ID grade	75.0	25.0	100.0

**Table 4:** ID grade and SCQ-cutoff crosstabulation females

			SCQ-cutoff		Total
			≤ 15	≥ 15	
ID grade	No ID	Count	1	2	3
		% within ID grade	33.3	66.7	100.0
	Mild	Count	0	1	1
		% within ID grade	0.0	100.0	100.0
	Moderate	Count	3	7	10
		% within ID grade	30.0	70.0	100.0
	Severe	Count	0	1	1
		% within ID grade	0.0	100.0	100.0
Total		Count	4	11	15
		% within ID grade	26.7	73.3	100.0

### Social Responsiveness Scale

The SRS scores from 28 patients were analysed. A total of 71% of the scores were in the severe range, and 25% were in the mild to moderate range. Only 4% were in the normal range. Total scores and all subscales were higher in females on both standardised and raw scores. The gender difference was significant only in the subscales of Social Awareness and Social Cognition. The total *T* score and the raw score of Social Awareness and Social Cognition also had between large and very large effect sizes on the differences between males and females. A total of 87% of the females’ and 54% of the males’ scores were within the severe range, 13% of the females’ and 38% of the males’ scores fell in the mild to moderate range and 8% of the males’ scores was in the normal range. All the SRS scores are summarised in Table 5.

**Table 5:** Social Responsiveness Scale scores

	Total (*n* = 28)	Females (*N* = 15)	Males (*N* = 13)	Significant *p* value (Cohen’s *d*)
Total *T* score (SD)	82.29 (12.63)	89.73 (9.88)	73.69 (9.77)	0.000 (1.63)
Social Awareness raw score (SD)	12.43 (2.73)	13.60 (2.53)	11.08 (2.36)	0.011 (1.03)
Social Cognition raw score (SD)	11.68 (5.36)	19.47 (5.00)	13.46 (3.82)	0.001 (1.35)
Social Communication raw score (SD)	27.93 (7.70)	29.60 (6.72)	26.00 (8.56)	0.233 (0.47)
Social Motivation raw score (SD)	13.07 (5.26)	14.47 (4.91)	11.46 (5.36)	0.137 (0.58)
Autistic Mannerisms raw score (SD)	21.14 (5.97)	21.73 (5.99)	20.46 (6.12)	0.585 (0.21)
Total raw score	91.32 (20.60)	98.87(17.65)	82.62(20.93)	0.038 (0.84)
SRS classification	*N*(%)[ratio]	*N*(%)	*N*(%)	
Normal (>60)	1(4)[0]	0	1(8)	^a^
Mild–moderate (60–75)	7(25)[0.35]	2(13)	5(38)	^a^
Severe (< 75)	20(71)[1.61]	13(87)	7(54)	0.096

^a^Not applicable due to small sample

### Vineland Adaptive Behavior Scale II

The VABS II scores from 24 patients were analysed. All the VABS II scores are summarised in Table 6. There was a difference in the adaptive behaviour composite score, between males and females, but the differences were not significant.

**Table 6:** Vineland Adaptive Behavior Scale II scores

	Total (*N* = 24^a^)	Females (*N* = 13)	Males (*N* = 11)	Significant *p* value (Cohen’s *d*)
VABS II standard score (SD)	56.88 (12.86)	52.85 (12.69)	61.64 (11.89)	0.094 (0.71)
Communication (SD)	57.92 (14.12)	54.38 (13.25)	52.09 (14.59)	0.193 (0.55)
Daily activities (SD)	61.79 (12.79)	62.69 (12.44)	60.73 (13.71)	0.719 (0.15)
Socialisation (SD)	62.54 (10.36)	59.46 (9.03)	66.18 (11.05)	0.123 (0.67)

^a^Four parents were not available for telephone interview

### Developmental Behaviour Checklist

The DBC scores from 27 patients were analysed and all scores were above the clinical cutoff (≥ 46). All the DBC scores are summarised in Table 7. The DBC did not have the same gender differences that we observed in the SRS and SCQ, except from the subscale Social Relating, where we found a strong tendency for more problems among the females (Cohen’s *d* 0.85).

**Table 7:** Developmental Behaviour Checklist scores

	Total (*N* = 27^a^)	Females (*N* = 15)	Males (*N* = 12)	Significant *p* value (Cohen’s *d*)
DBC total percentiles (SD)	84.44 (13.19)	83.73 (13.87)	85.33 (12.83)	0.759 (0.12)
Disruptive/antisocial percentiles (SD)	85.63 (16.24)	83.47 (18.45)	88.33 (13.26)	0.433 (0.30)
Self-absorbed percentiles (SD)	80.67 (12.47)	80.53 (11.89)	80.83 (13.68)	0.953 (0.02)
Communication disturbance percentiles (SD)	75.93 (20.75)	73.73 (20.76)	78.67 (21.33)	0.551 (0.23)
Anxiety percentiles (SD)	61.11 (28.28)	62.53 (29.15)	59.33 (28.31)	0.776 (0.11)
Social Relating percentiles (SD)	42.96 (24.82)	51.60 (25.28)	32.17 (20.33)	0.036 (0.85)

^a^One parent did not correctly fill out the questionnaire

### Effect of gender when controlling for developmental level and behavioural problems

To determine the impact of gender on the SCQ score when controlling for developmental level (VABS II standard score) and amount of emotional and behavioural problems (DBC total score), a linear regression was conducted with the total SCQ score as the dependent variable. Measuring IQ in individuals with SMS is known to be problematic due to their behavioural characteristics. Therefore, we use data from the VABS II as a proxy for developmental level.

When gender, VABS II and DBC were entered as covariates, we obtained a highly significant model of the SCQ score (*R*^2^ = 0.60, *F* = 8.8, *p* = 0.0008). Only gender had an independent contribution on the model (*β* = 0.70, *p* = 0.0003); VABS II (*β* = − 0.13, *p* = 0.44) and DBC (*β* = − 0.16, *p* = 0.31) had no independent contribution.

A similar linear regression was conducted with the SRS total raw score. When gender, VABS II and DBC were entered as covariates, we still obtained a significant model of the SRS total raw score (*R*^2^ = 0.46, *F* = 5.1, *p* = 0.010). Both gender (*β* = 0.46, *p* = 0.022) and DBC (*β* = 0.48, *p* = 0.013) contributed to the model. VABS II (*β* = 0.04, *p* = 0.836) had no independent contribution. More details from the models are displayed in Table 8.

**Table 8:** Regression model summary

	SCQ total			SRS raw score		
*Factors*	*B*	*p*	95%	*B*	*p*	95%
Constant	12.44	0.204	− 7.38/32.25	− 21.43	0.615	− 109.37/66.50
Gender	8.30	0.0003	4.34/12.24	20.95	0.022	3.42/38.48
VABS II	− 0.08	0.44	− 0.21/0.10	0.96	0.836	− 0.74/0.61
DBC	− 0.06	0.31	− 0.25/0.08	− 0.07	0.013	0.23/1.68
Model’s *R*^2^	0.60			0.46		
Model’s *p* value	0.0008			0.010		

*B* unstandardized B, *Sig* significant level, *95%* confidence interval for B for each factor

## Discussion

This study explored a number of ASD symptoms across gender in a Scandinavian SMS sample. The approximately three females per male above the SCQ cutoff is exactly the opposite of what we would expect to find in a sample of idiopathic ASD. It is particularly in the social domain of ASD that females with SMS differ substantially from females with other aetiological pathways to ASD.

The reversed gender ratio of ASD symptoms identified in this study cannot be explained by differences in neither developmental level nor in the amount of emotional and behavioural problems. The clinical diagnoses of intellectual disability differ between the genders, and we found a tendency for poorer development in females (VABS II total 53) than males (VABS II total 62), but this difference was not significant. In the regression model, the VABS II score did not have an independent contribution to the SCQ score. Emotional and behavioural problems, as measured with the DBC, did not differ between the sexes. In the regression model of the SRS, we found that DBC contributed in addition to gender. This probably indicates that the SRS is more sensitive to behavioural problems than the SCQ is [32, 45]. The SRS places a heavier emphasis on the reciprocal social interaction trait in ASD, whereas the SCQ places a similar emphasis on all three ASD domains [45].

Neither Oliver [16] nor Vignoli [17] found any significant gender differences in ASD symptomatology in other rare genetic syndromes such as cri du chat syndrome, Cornelia de Lange syndrome, Prader Willis syndrome or tuberous sclerosis complex.

We wanted to investigate whether a difference in ASD symptomatology could be the result of females having more severe phenotypes than males and if it could be linked to levels of ID or whether the emotional and behaviour problems in SMS affected gender differences. We found a strong tendency for lower degrees of ID in females than in males, but the difference was not significant. But as the difference is approaching significance (0.07), it would be interesting to investigate further if there could be a real gender difference in ID levels in SMS. As mentioned before, the accuracy of our ID levels is questionable and therefore not used to draw any conclusions. In general, administering formal psychometric assessments is often reported to be very difficult with people with SMS, due to the maladaptive behaviours, sleep disturbance and difficulties in expressive language skills [46].

The observed gender differences in ASD do not seem to be related to the main genetic mechanisms for SMS. The RAI1 mutations, associated with less severe SMS phenotype, were more frequent in females (3/20%) with more ASD symptoms than in males (0/0%) who had less ASD symptoms. The group of individuals with RAI1 mutations was too small to be tested as a separate subgroup in any of the analyses.

Current research suggest that female protective factors are more important than particular male-linked risk in explaining the male bias in ASD, but the mechanisms behind such female protection are not established [4, 6]. Whatever the female protective factor turns out to be, the current data suggest that it is not present in females with SMS.

We found three other papers presenting gender differences in SMS [19, 24, 30]. In the study from Edelman et al. [30], the authors found some gender differences, with the females showing more problems. Most of them were somatic (myopia, cold hands and feet, eating/appetite problems and possible hypersensitivity (problems finding shoes to fit)), but they also found that females had a significantly higher frustration with communication level. Neither of the questionnaires used in our study found a significant gender difference regarding communication, but a more thorough investigation of communication profiles in this syndrome would be beneficial both to investigate the gender difference more and to propose possible interventions. The study by Laje et al. [19] indicated an absence of the usual gender difference regarding ASD measured with SRS but not SCQ. In our study, we find gender differences both in the SRS and in the SCQ measure, both showing more problems among the females. It is particularly the social domain of ASD that has an unusual male/female ratio. Females with SMS have significantly more social problems than males. We did not find any difference in repetitive behaviour. Laje et al. [19] found a gender difference, favouring the females, in two subscales on the SRS but not in the total raw score or on the SCQ. In our study, we found a gender difference in ASD symptomatology, but neither in our study nor in the study by Laje et al. [19] could this difference be explained by differences in other traits in the syndrome. IQ level, adaptive behaviour and general emotional and behaviour problems have been investigated. A more thorough investigation of gender differences in adaptive behaviour profiles and the emotional and behaviour problems would be beneficial, alongside further molecular research regarding possible sexually dimorphic processes in SMS.

### Limitations

Assigning a formal diagnosis of ASD to individuals with a known genetic syndrome is a matter of some debate [15]. In the current study, we only used the SCQ and the SRS as a measure of the number of ASD symptoms; we did not observe or use diagnostic instruments such as ADI-R or ADOS. Hence, we do not have data on how many actually fulfil the criteria for an ASD diagnosis.

Measuring IQ in individuals with SMS, as mentioned earlier, is known to be problematic due to their behavioural characteristics. Therefore, data from the VABS II were used as a proxy for developmental level. Even though VABS II cannot substitute a formal psychometric assessment such as IQ tests, consistency has been demonstrated between formal IQ tests and the VABS II [41]. In this study, we used developmental level instead of intellectual level/disability in most of our analysis, due to the fact that we ourselves did not collect the ID levels and could not guarantee for their validity.

## Conclusion

We found a clear reversed gender difference in the number of ASD symptoms in persons with SMS. This female bias in ASD symptoms is not explained by differences in the developmental level or the amount of emotional and behavioural problems. The deletion that is known to cause SMS is located on chromosome 17 (17p11.2), and there is no known reason to expect gender differences in any traits in this autosomal condition. The finding of a clear gender difference is therefore notable, and the mechanisms behind this require further study. A previous study found a sexually dimorphic phenotype in eating behaviour in mice with loss of RAI1 functions [24]. Whether this is related to our finding should be explored. Knowledge about the biological underpinnings of the reversed ASD gender ratio may be of relevance to understand gender differences in other biological pathways to ASD. The female protective factors assumed to explain the male bias in ASD seems to be lacking in SMS.

## Abbreviations

ADI-R: Autistics Diagnostic Interview-Revised
ADOS: Autism Diagnostic Observation Schedule
ASD: Autism spectrum disorder
DBC: Developmental Behaviour Checklist
ID: Intellectual disability
RAI1: Retinoic acid induced 1
SCQ: Social Communication Questionnaire (SCQ)
SMS: Smith-Magenis syndrome
SRS: Social Responsiveness Scale
VABSII: Vineland Adaptive Behavior Scale II
